# An analysis of chronic kidney disease as a prognostic factor in pediatric cases of COVID-19

**DOI:** 10.1590/2175-8239-JBN-2020-0208

**Published:** 2021-03-05

**Authors:** Bárbara Caroline Dias Faria, Luiz Gustavo Guimarães Sacramento, Carolina Sant’ Anna Filipin, Aniel Feitosa da Cruz, Sarah Naomi Nagata, Ana Cristina Simões e Silva

**Affiliations:** 1 Universidade Federal de Minas Gerais, Faculdade de Medicina, Belo Horizonte, MG, Brasil.; 2 Universidade Federal de Minas Gerais, Faculdade de Medicina, Departamento de Pediatria, Belo Horizonte, MG, Brasil.

**Keywords:** Coronavirus Infections, Renal Insufficiency, Chronic, Child, Prognosis, Infecções por Coronavirus, Insuficiência Renal Crônica;Criança, Prognóstico

## Abstract

Advanced age is a risk factor for severe infection by acute respiratory syndrome coronavirus 2 (SARS-CoV-2). Children, however, often present with milder manifestations of Coronavirus Disease 2019 (COVID-19). Associations have been found between COVID-19 and multisystem inflammatory syndrome in children (MIS-C). Patients with the latter condition present more severe involvement. Adults with comorbidities such as chronic kidney disease (CKD) are more severely affected. This narrative review aimed to look into whether CKD contributed to more severe involvement in pediatric patients with COVID-19. The studies included in this review did not report severe cases or deaths, and indicated that pediatric patients with CKD and previously healthy children recovered quickly from infection. However, some patients with MIS-C required hospitalization in intensive care units and a few died, although it was not possible to correlate MIS-C and CKD. Conversely, adults with CKD reportedly had increased risk of severe infection by SARS-CoV-2 and higher death rates. The discrepancies seen between age groups may be due to immune system and renin-angiotensin system differences, with more pronounced expression of ACE2 in children. Immunosuppressant therapy has not been related with positive or negative effects in individuals with COVID-19, although current recommendations establish decreases in the dosage of some medications. To sum up with, CKD was not associated with more severe involvement in children diagnosed with COVID-19. Studies enrolling larger populations are still required.

## Resumo

A idade avançada atua como fator de risco para infecção grave por coronavírus 2 da Síndrome de Angústia respiratória aguda (Sars-CoV-2). Em contrapartida, crianças comumente apresentam curso clínico mais brando da Doença do coronavírus 2019 (covid-19). Entretanto, há associação entre covid-19 e Síndrome Inflamatória Multissistêmica em Crianças (MIS-C). Nesses casos, os pacientes apresentam pior evolução. Além disso, em presença de comorbidades, como doença renal crônica (DRC), a população adulta cursa com pior prognóstico. Este estudo objetivou, por meio de uma revisão narrativa, avaliar se a DRC contribuiu para pior prognóstico na covid-19 em pacientes pediátricos. Os estudos analisados mostraram ausência de casos graves, nenhum óbito e rápida recuperação tanto em crianças com DRC quanto nos pacientes pediátricos previamente hígidos. Contudo, alguns pacientes com MIS-C necessitaram de internação em unidade de terapia intensiva e houve registro de óbitos, mas não foi possível relacionar a ocorrência de MIS-C com presença de DRC. Por outro lado, nos adultos, a DRC associou-se a risco aumentado de infecção grave por Sars-CoV-2, com maior índice de óbitos. As discrepâncias entre os grupos etários podem se dever a diferenças no sistema imunológico e no sistema renina-angiotensina, com maior expressão de ECA2 nas crianças. Nos pacientes em imunossupressão, ainda não foi determinado se este tratamento apresenta efeitos benéficos ou maléficos no curso da covid-19, com atual recomendação de se reduzir a dose de alguns medicamentos. Concluindo, não foi possível associar a DRC em crianças a piores prognósticos de covid-19, sendo necessários estudos com amostras mais significativas.

## Introduction

December of 2019 marked the start of the dissemination of a new infectious disease called Coronavirus Disease 2019 (COVID-19) in the Chinese city of Wuhan, caused by the severe acute respiratory syndrome coronavirus 2 (SARS-CoV-2)^1^. In light of its rapid global dissemination, the World Health Organization (WHO) elevated the disease to the category of a pandemic on March 11, 2020^2^. SARS-CoV-2 is a severe threat to public health that has produced a significant number of deaths and tens of millions of confirmed cases of infection throughout the world. On August 30, 2020, when this paper was being finalized, 25.03 million individuals had been diagnosed with the disease with an incidence rate of 3,211 cases per million population and a mortality rate of 3.4%, adding up to 843,158 deaths and 108.17 deaths per million population^3^.

Six months after the first case of COVID-19 was recorded in Brazil, the 28^(th)^ Special Epidemiological Newsletter indicated that 316,814 Brazilians infected with SARS-CoV-2 by August 22, 2020 had been hospitalized, of which 7,436 (2,3%) were aged 0-19 years. Children were further subdivided into groups of subjects aged less than a year with 1,934 cases, aged 1-5 years with 1,862 cases, and aged 6-19 years with 3,642 cases. The death toll read 111,258, of which 759 (0.6%) were aged 0-19 years. In the subgroups of children, 248 deaths of individuals aged less than a year, 123 deaths of individuals aged 1-5 years, and 388 deaths of individuals aged 6-19 years were recorded. About a quarter of the deaths (29,522) were recorded among individuals aged 20-59 years, while 80,977 individuals aged 60 years or older (75%) died of the disease^4^.

Although pediatric patients apparently experience milder forms of COVID-19, multisystem inflammatory syndrome in children (MIS-C) has been reported as a possible complication of infection by SARS-CoV-2^5^.

Middle-aged and elderly subjects are at higher risk of developing severe acute respiratory syndrome (SARS), experiencing complications, and dying. There is consensus in the literature about the fact that advanced age and underlying disease - including chronic kidney disease (CKD)^6^ - are important risk factors for severe infection by SARS-CoV-2^1^. However, we still lack conclusive information on the possible role CKD may have in the development of COVID-19 in pediatric patients.

### Objectives

This review looked into literature on pediatric patients with CKD to verify whether they were more prone to developing more severe symptoms when diagnosed with COVID-19 compared to children without CKD and adults with CKD.

## Methods

This narrative review included papers listed in the following databases: PubMed, MEDLINE, Springer, WHO R&D Blueprint, BVS, and Cochrane. Keywords “Covid-19”, “Sars-CoV-2”, “Covid-19 and Child”, “Chronic Kidney Disease”, “Pediatric Kidney Transplantation”, “Chronic Kidney Disease dialysis”, “Children”, “Angiotensin-Converting Enzyme 2”, “Kawasaki Disease” were used. Studies reporting data on pre-defined outcomes related to infection by SARS-CoV-2 in children with CKD, children without CKD, and adults with CKD were included.

### Ethics

This study is a literature review and, as such, does not require submission to or approval by an Ethics Committee, as set out in Resolution 466/12 of the National Health Council (CNS, Brazilian acronym). However, established ethical principles concerning the legitimacy, secrecy, and private nature of the information provided herein were complied with when needed.

## Results

### Pediatric patients without CKD

Haiyan et al. reviewed the cases of 36 children aged 1-16 years tested positive for COVID-19. Seventeen (47%) patients had mild clinical symptoms, ten (28%) were asymptomatic, and seven (19%) had symptoms consistent with acute upper airway infection. Nineteen (53%) had moderate symptoms and mild pneumonia. None had severe disease. The following symptoms were described: fever (36%), dry cough (19%), headache (8%), sore throat (6%), vomiting or diarrhea (6%), dyspnea or tachypnea (3%), and pharyngeal congestion. All children were treated with aerosolized interferon-alpha twice a day, 14 (39%) were prescribed lopinavir-ritonavir oral solution twice a day, and six (17%) required supplemental oxygen. The patients were hospitalized for 14 days on average^7^.

A study performed in Northern China reviewed the cases of 31 children aged six months to 17 years who were hospitalized after testing positive for SARS-CoV-2 with RT-PCR. None had comorbidities. Four patients (13%) were asymptomatic, 13 (42%) had mild symptoms, and 14 (45%) were categorized as common type. None developed severe disease. Twenty (65%) had fever lasting from one to nine days. One had high fever (> 39.1ºC), nine had moderate fever (38.1-39.0ºC), and ten had low fever (37.3-38.0ºC); 14 (45%) had cough; three (10%) had fatigue; three (10%) had diarrhea. Other symptoms including sore throat, coryza, dizziness, headache, and vomiting were rarely reported. Kidney dysfunction and blood glucose level alterations were not found. Fourteen children had alterations in their chest computed tomography scans, of which nine had nodules and uneven ground-glass opacity. Most of the patients (n = 29) were prescribed antiviral therapy: ten were treated with interferon (nebulized or pulverized for oral administration, 6-10 days); one individual was treated with oseltamivir alone (oral, twice a day, two days); the other patients were given combined therapy with interferon (nebulized or oral spray, 2-24 days), oseltamivir phosphate (oral, twice a day, five days), ribavirin (intravenous, 9-13 days), Arbidol (oral, 6-16 days), lopinavir/ritonavir (oral, twice a day, 7-18 days). On day 18, one patient had elevated transaminase levels without other adverse reactions. Antibacterial drugs were prescribed to six children for 5-11 days. Two children were treated with intravenous infusions of gamma globulin (379 mg/kg/day and 278 mg/kg/day) for four days. Eight children were given treatment for symptoms based on traditional Chinese medicine. Between Days 7 and 23, 25 children tested negative; six remained hospitalized in short-term isolation; one subject was not discharged for presenting acute suppurative tonsillitis^8^.

The studies mentioned in this section are listed in Table 1.

**Table 1:** Pediatric patients without CKD

Author	Study design	Population	Summary of findings
Haiyan et al., 2020	Cohort study	36	- No child had severe COVID-19 symptoms or died;
			- Study limitation: small population.
Wang et al., 2020	Retrospective study	31	- No child had severe symptoms or died;
			- Pediatric patients recovered quickly and had negative T-PCR tests for SARS-CoV-2 within 7-23 days on average.

### Kawasaki-Like disease and hyperinflammatory syndrome in pediatric patients

A Brazilian study followed 79 children admitted to 19 pediatric intensive care units and found that 41% had comorbidities prior to hospitalization, 28% of which were neuromuscular conditions - predominantly non-progressive encephalopathies, while chronic respiratory diseases, cancer and blood diseases, congenital heart defects, and malnutrition combined were seen in 27% of the patients. Diabetes, prematurity, chronic liver disease, and obesity were observed in 18% of the patients. In this study, Prata-Barbosa et al. looked into the predictive factors tied to severe forms of COVID-19. The patients were subdivided into groups to elicit correlations between sex, ethnicity, age of less than one year, and comorbidities (32-41%) to severity of involvement, denoted by the prescription of invasive mechanical ventilation (IMV). Results with a confidence interval (CI) of 95% failed to find direct associations between male sex (p = 0.32), non-Caucasian ethnicity (p = 0.80), and age of less than one year (p = 0.64) with use of IMV; however, the study revealed that the presence of comorbidities alone (p = 0.01) was significantly associated with severe involvement. The patients included in the study were aged between one month and 19 years, resulting in a mean age of four years; 43 (54%) were boys and 36 (46%) were girls. Nineteen (24%) of the hospitalized patients were aged less than one year; five (26%) had comorbidities and three (16%) required IMV. Fourteen patients (18%) required IMV. Ten of them (71%) had comorbidities and in none was CKD described as a chronic condition. The study found that ten children (12,7%) had MIS-C, and that two of them (20%) had comorbidities and one (10%) required IMV; their main symptoms were fever (n=8; 80%), tachypnea (n=6; 60%), vomiting (n=6; 60%), and malaise (n=6; 60%). The primary clinical manifestation in individuals with MIS-C was Kawasaki-like disease, seen in six patients (60%). Sixty-nine children (87,3%) did not develop MIS-C; 30 of them (43%) had comorbidities and 13 (19%) required IMV; their main symptoms were fever (n=51; 75%); cough (n=36; 53%); tachypnea (n=33; 49%); and low SpO2 (< 92%) (n=19; 28%). Two deaths (3%) of individuals without MIS-C and with comorbidities were recorded. The children with comorbidities were generally older, with a mean age of 7.5 years; they also required more IMV (31% *vs.* 9%, p = 0.01) and were more frequently diagnosed with acute respiratory distress syndrome (ARDS) (25% *vs.* 4%, p = 0.01)^9^.

Feldstein et al. analyzed the cases of 186 patients aged 0-21 years diagnosed with MIS-C, which incidence increased when COVID-19 rates were decreasing in the United States. Seventy percent of the patients (n=131) had tested positive in RT-PCR tests, antibody tests or both for infection by SARS-CoV-2. The remaining 55 (30%) had been in contact with individuals with COVID-19. Seventy-three percent (n=135) had been healthy individuals, and comorbidities affecting the other patients were not thoroughly described. Thirty-five patients (19%) were non-Hispanic whites, 46 (25%) were non-Hispanic blacks, 9 (5%) were of other non-Hispanic ethnicities, 57 (31%) were Hispanic or Latinos, and 41 (22%) were of unknown ethnicity. A total of 132 patients (71%) had involvement of at least four systems, the more common of which were the gastrointestinal (92%), cardiovascular (80%), hematologic (76%), mucocutaneous (74%), and respiratory (70%) systems. Eighty percent of the patients (n=148) required hospitalization at an intensive care unit, one fifth (n=37) needed IMV, and eight individuals (4%) received extracorporeal membrane oxygenation (ECMO). In the subgroup with MIS-C, 74 patients (40%) had fever for at least five days and presented four or five traits similar to Kawasaki disease or two or three traits similar to Kawasaki disease with additional lab workup and echocardiogram findings. On May 20, 2020, 130 patients (70%) were discharged, four (2%) died, and the rest of the group remained in hospital^10^.

The studies mentioned in this section are listed in Table 2.

**Table 2:** Kawasaki-like disease and hyperinflammatory syndrome in pediatric patients

Author	Study design	Population	Summary of findings
Arnaldo Prata-Barbosa et al., 2020	Multicenter prospective study	79	- Most patients who needed IMV had comorbidities;
			- Kawasaki-like disease was the main clinical presentation in patients with MIS-C;
			- Gastrointestinal symptoms were more prevalent in patients with MIS-C;
			- Sixty-nine children did not develop MIS-C; 30 had comorbidities and 13 required IMV;
			- Two patients died;
			- Children with comorbidities were older, required more oxygen therapy, and were more frequently diagnosed with ARDS.
			- None of the patients had CKD.
Feldstein et al.	Multicenter study	186	- Comorbidities were not described in detail;
			- The incidence of MIS-C increased after peaks in the number of cases of COVID-19;
			- One hundred and thirty-two patients had involvement in at least four systems;
			- One hundred and forty-eight were admitted to an intensive care unit, 37 were on IMV, and eight on ECMO;
			- One hundred and thirty were discharged, four died, and the other patients were still hospitalized at the end of the study.

### Pediatric patients with CKD

A study performed in Spain enrolled 16 children under the age of 18 years previously diagnosed with CKD tested positive for infection by SARS-CoV-2 in RT-PCR tests. Ten patients had pre-dialysis CKD, three were on hemodialysis, and three had undergone kidney transplantation. By way of symptoms, 62.5% of the children had coughs and/or rhinorrhea, 50% had fever, 25% had gastrointestinal symptoms, and three (19%) were asymptomatic. Data on lymphopenia were available only for 12 patients, and four (30%) had the condition. Eight of the 16 patients were hospitalized, but none required admission to a pediatric intensive care unit. Nine children had been on immunosuppressants previously (three transplant patients, one on chronic hemodialysis with vasculitis, four with nephrotic syndrome, and one with IgA nephropathy). Immunosuppressant therapy was interrupted or dosages were decreased in four children. Five patients were prescribed hydroxychloroquine and one patient had been first given lopinavir-ritonavir, which resulted in adverse gastrointestinal effects, and was later converted to therapy with hydroxychloroquine. The patients recovered fully 19 days after diagnosis on average, and no death was recorded^1^.

The European Rare Kidney Disease Reference Network started a study involving 16 centers from 11 countries. The study included 18 children aged 0-19 years on immunosuppressants diagnosed with COVID-19; 11 patients had previously undergone kidney transplants. None of the patients had dyspnea or required admission at an intensive care unit (ICU)^11^.

Members of the European Reference Network on Pediatric Transplantation from the Padova University Hospital (Italy) and the La Paz University Hospital (Spain) sent out a questionnaire to other members in order to assess the impact of COVID-19 in pediatric transplants performed in Europe. Eighteen centers from 11 countries answered the questionnaire, in which two kidney transplant candidates and two transplant patients were cited as having been diagnosed with COVID-19. All had mild clinical signs and none required admission at an intensive care unit or changes to immunosuppressant therapy^12^.

A case report published by Bush et al. described the case of a 13-year-old who contracted COVID-19 five years after having a kidney transplant. The patient did not develop complications or require changes to immunosuppressant therapy, and recovered rapidly^13^.

In the United Kingdom, five children with CKD stages IV or V tested positive for infection by SARS-Cov-2. None died^14^.

The studies mentioned in this section are listed in Table 3.

**Table 3:** Pediatric patients with CKD

Author	Study design	Population	Summary of findings
Melgosa et al., 2020	Retrospective study	16	- On average, the children recovered fully 19 days after they had been diagnosed with COVID-19. None died.
Marlais et al., 2020	Correspondence	18	- Patients had mild manifestations of COVID-19 and did not require admission to an intensive care unit. None died.
Doná et al., 2020	Brief communication	14	- Patients had mild manifestations of COVID-19 and did not require admission to an intensive care unit. None died.
Bush et al., 2020	Case report	1	- A 13-year-old with COVID-19 after a kidney transplant had mild symptoms, progressed without complications, and recovered quickly.
Plumb et al., 2020	Retrospective study	5	- Patients with CKD stage IV and V recovered filly from infection by SARS-CoV-2.

### Adult patients with ckd

A study enrolled 12 kidney transplant patients aged 29-66 years diagnosed with COVID-19 based on positive RT-PCR tests for SARS-CoV-2. The most common symptoms were fever, cough, and dyspnea, seen in 75%, 75%, and 41.7% of the patients, respectively. A third of the patients had leukopenia. All had been on immunosuppressants, and dosages were decreased based on the protocols in effect at the center in which the study was carried out. The patients were prescribed hydroxychloroquine 400 mg, lopinavir-ritonavir 400/100 mg twice a day, and intravenous antibiotics. Ten were admitted to an intensive care unit and eight died of severe pneumonia caused by COVID-19 and acute respiratory distress syndrome^15^.

In a meta-analysis, Brandon Michael Henry and Giuseppe Lippi showed that four studies analyzed separately did not report CKD as a significant predictor for severe COVID-19 in adults. However, the combined data revealed a significant association between CKD and severe COVID-19^6^.

The studies mentioned in this section are listed in Table 4.

**Table 4:** Adult patients with CKD

Author	Study design	Population	Summary of findings
Abrishami et al., 2020	Case series	12	- Ten of 12 kidney transplant patients were admitted at an intensive care unit and eight died.
Henry, Lippi, 2020	Meta-analysis	1.389, of which 273 had CKD	- CKD was associated with increased risk of severe infection by SARS-CoV-2.

## Discussion

In the context of the SARS-CoV-2 pandemic, there is mounting concern over the infection of pediatric patients with CKD, since in adults the disease is a risk factor for severe involvement. Specialists suggested that children with CKD might be at higher risk during the pandemic on account of immunosuppressant therapy and greater exposure to hospitals and health care clinics, since they require hemodialysis and other therapies^16^.

Few papers have been published about children with CKD who become infected by SARS-CoV-2, possibly because children account for only 1-5% of the cases of COVID-1^17^. and there is little epidemiological data on pediatric CKD. The prevalence of CKD stage V in the United States in 2016 was 104 per million patients aged 0-21 years^18^. In contrast, prevalence among young adults aged 22-44 years was approximately 967 per million population^18^. and in the group aged 45-64 years prevalence was approximately 3,883 per million population^18^. The prevalence of CKD stage V clearly increases with age.

In the analyzed studies, the most common symptoms among pediatric patients with CKD were cough, fever, gastrointestinal symptoms, and symptoms consistent with acute upper airway infection, as also seen in children without CKD. Although children with CKD stay in hospital for longer, the disease played out similarly as it did in healthy children, with mostly mild symptoms and good progression.

Evidence suggests the existence of an association between infection by SARS-CoV-2 and MIS-C based on temporal relations, positive COVID-19 tests in most patients with MIS-C, and hyperinflammatory manifestations similar to what is seen in adult COVID-19 patients^10^^,^ 19 ^-^ 21. MIS-C has been described as an unusual complication of COVID-1^10^. that manifests weeks after infection with greater prevalence observed after peaks in COVID-19 cases. The condition has been associated with more severe involvement and greater need of IMV. However, the reasons why only some children and adolescents develop MIS-C are unclear. A potential explanation is based on age-related differences, which might yield different probabilities of exposure to SARS-CoV-2 or differences in the nasal expression of ACE2^10^^,^ 22. Another possibility is that children genetically susceptible to Kawasaki disease might present decreased expression of membrane-bound ACE2^23^. Once they are infected with SARS-CoV-2, the expression of ACE2 would be more significantly deregulated by TNF-α, leading to inflammation and development of Kawasaki-like disease^23^. However, an association between CKD and MIS-C has not been described. It should be noted that the epidemiological manifestation of Kawasaki disease associated with COVID-19 is different from the norm. In general terms, the condition is more prevalent during early childhood in individuals of Asian descent, while in subjects with SARS-CoV-2 it occurs in older, previously healthy pediatric patients of African and Hispanic descent^5^.

Comparisons between pediatric and adult patients with CKD revealed that adult individuals with COVID-19 developed more severe disease. The most common symptoms observed in these patients were fever, cough, dyspnea, severe pneumonia, and acute respiratory distress syndrome. Most of the adult patients had to be admitted to an intensive care unit and many eventually died. An association has been found between CKD and severe COVID-19 in adult patients. The same cannot be said of pediatric patients with CKD.

A few ideas have been considered to explain the causal link between the different forms and manifestations of COVID-19 in adults and children, since prognosis is connected not only to preexisting comorbidities, but also to physiological oscillations in the molecules of the renin-angiotensin system (RAS) that occur during the course of life^24^. Zhou et al. confirmed that SARS-CoV-2 uses angiotensin converting enzyme 2 (ACE2) to penetrate host cells^25^. ACE2 is the first known angiotensin converting enzyme (ACE) homologue, with 40% identity and 60% similarity. It carries an apparent signal peptide, one active site of metalloproteinase and one transmembrane domain^26^^,^ 27. ACE2 is also present in other sites and cells, including alveolar cells and lymphocytes, which may explain lung involvement and lymphopenia in individuals with COVID-19^28^.

In the RAS, ACE promotes the conversion of angiotensin I (Ang I) into angiotensin II (Ang II), which binds to the AT1 receptor. This axis, known as the classical axis, produces vasoconstriction and proinflammatory and pro-oxidative effects. By its turn, ACE2 converts Ang II into Angiotensin-(1-7) [Ang-(1-7)]^28^. The main effects of Ang-(1-7) are mediated by a G-protein coupled receptor called Mas (MasR)^29^, which triggers vasodilation, antioxidant and anti-apoptotic effects, and inhibition of inflammatory response and fibrosis^28^^,^ 30 ^,^ 31. Therefore, the ACE2/Ang-(1-7)/MasR axis, also known as the counter-regulatory axis, acts in opposition to the ACE/AngII/AT1R axis^28^, as both play an important role in the regulation of the immune system^30^^-^ 32.

In regard to the anti-inflammatory effects of the counter-regulatory axis, studies performed with animal models suggested that its activation leads to decreased expression of proinflammatory cytokines interleukin 1 (IL-1)^30^^,^ 33 ^-^ 37, interleukin 5 (IL-5)^30^^,^ 38. interleukin 6 (IL-6)^30^^,^ 33 ^,^ 39 ^-^ 46. tumor necrosis factor alpha (TNF-α)^30^^,^ 47, and monocyte chemotactic protein-1 (MCP-1)^30^^,^ 46. The activation of this axis also decreases the production of chemokine^30^^,^ 36 ^,^ 41 ^,^ 46 ^,^ 48 ^-^ 50, and increases the production of interleukin 10 (IL-10), anti-inflammatory cytokine^30^^,^ 36 ^,^ 38 ^,^ 50, during inflammation. Besides, studies with animal models found that the counter-regulatory axis acts in the modulation of the expression of enzyme cyclooxygenase-2^30^^,^ 33 ^,^ 34 ^,^ 51 ^,^ 52, decreasing prostaglandin production.

COVID-19 apparently causes, at least in part, imbalances in the RAS by negatively regulating ACE2, thereby exacerbating the ACE/AngII/AT1R axi^53^, and producing predominantly proinflammatory effects. This imbalance is the outcome of an apparent paradox related to the bioavailability of ACE2: either (A) infected individuals have higher levels of ACE2 and are able to support the exacerbated consumption of this enzyme and neutralize the deleterious effects caused by low ACE2 levels; or (B) individuals with naturally lower ACE2 levels cannot activate the anti-inflammatory pathway of the RAS, thereby exacerbating the ACE/AngII/AT1R axis^54^. By its turn, this exacerbation, via endothelial dysfunction and cytokine storm, induce acute lung injury^30^.

In general terms, children have higher ACE2 expression levels than adult^31^^,^ 55, and tend to present responses consistent with case A described above, with cytokine storm and severe COVID-19 becoming less probable events. Furthermore, Bunyavanich et al. showed that children aged less than ten years have lower ACE2 expression levels in the nasal epithelium - one of the main entry points of SARS-CoV-2 - than older children and adults^22^^,^ 56, which explains the lower incidence of infection in this age group. In addition, estrogen and testosterone levels decrease with age, thereby increasing plasma renin activity and changing the balance between the RAS axes^57^. Therefore, a natural negative regulation of the ACE2/Ang-(1-7) axis occurs in the elderly^58^, making them more susceptible to severe COVID-19. 

The milder forms of viral diseases seen in children may also be due to the fact that they have relatively immature immune systems^56^^,^ 59, with low T and B cell levels, lesser Th1 and IFN type 1 inflammatory response, and greater Th2 and Th17 inflammatory response^56^^,^ 60 ^,^ 61. Furthermore, the high level of regulatory T cells in children may protect them against severe manifestations of COVID-19^56^^,^ 62. By their turn, adults usually present greater levels of Th1 inflammatory response, as verified in infections by SARS. Greater magnitude Th1 response is closely related to severe diseas^56^^,^ 63 ^,^ 64, and, due to the significant homology between its viral sequence and SARS-CoV-2, it is likely that this mechanism also occurs in COVID-19^56^. One should notice, however, that we still lack evidence to define the causes of milder disease in children. The hypotheses currently considered require confirmation from future studies.

The effects of immunosuppressant therapy in individuals infected with SARS-CoV-2 have been discussed in the literature. Immunosuppression is one of the cornerstones in the treatment of kidney disease. Discontinuing therapy may significantly compromise the health of individuals with kidney disease and the management of their underlying condition, which may become more harmful than the viral infection itself^65^.

Immunosuppression may facilitate contamination and dissemination of the virus in one’s body, ultimately producing more severe cases of COVID-19^66^. Data from the Centers for Disease Control and Prevention (CDC) collected between February and April 2020 showed that among children with comorbidities diagnosed with COVID-19, immunocompromised individuals were the more severely affected^65^. Among children with CKD, immunocompromised patients include children with stage IV or V disease, subjects on hemodialysis, and individuals on immunosuppressants, including steroids, calcineurin inhibitors, cyclophosphamide, mycophenolic acid, and rituximab^65^. Calcineurin inhibitors are prescribed to transplant patients and cause significant decrease in adaptive immune response, which potentially increases the chances or uncontrolled virus dissemination^66^. In vitro studies have shown that non-immunosuppressive derivatives of cyclosporine may decrease the levels of viral N protein, an element in viral replication. One might speculate that such derivatives may be used instead of calcineurin inhibitors within the context of the SARS-CoV-2 pandemic^66^.

Some authors have suggested that immunosuppression may have a role in the treatment of patients with COVID-19^67^^,^ 68. Since one of the pathophysiological mechanisms of the disease is a state of hyperactivation of the immune system, particularly with the exacerbated activation of T cells, the anti-inflammatory effects of immunosuppressants might decrease immune response, and consequently ameliorate lung injuries and reduce the severity of the disease^67^. Xu et al. analyzed post mortem biopsy specimens and found high levels of CD4 T cells with CCR6+ and Th17 in lung tissue^68^. The severity of lung injury may be connected, among other factors, to increased T cell activity in the organ. Additionally, lymphopenia in patients with severe COVID-19 brings up the possibility of hyperactive defense cells being sequestered to the lungs, thus decreasing their circulating levels. Chronic immunosuppressant therapy and compromised T cell function might produce a protective effect against the complication arising from the disease^67^.

Prescribed medications include tacrolimus and cyclosporine, which decrease the production of IL-2, a cytokine that modulates T cell proliferation and maturation, and tacrolimus and mycophenolic acid, which inhibit IL-17 and Th17 lymphocytes, thus decreasing stimuli to produce IL-6 and IL-8^67^. Therefore, another consequence of immunosuppressant therapy is decreased cytokine release, which by its turn may prevent the occurrence of cytokine storms characteristically seen in patients with COVID-19 and thus yield milder symptoms^13^.

The continuation of immunosuppressant therapy and the initiation of such therapy in clinically eligible patients with CKD are warranted^65^^,^ 66 ^,^ 67 ^,^ 69. The general recommendation for patients suspected or diagnosed with infection by SARS-CoV-2 is to reduce immunosuppression to safer levels that allow the management of the underlying condition and decrease the risk of infection^66^. More studies are needed to define the order of magnitude of such decrease in immunosuppressant therapy. In this context and in all other related medical decisions, patient individual characteristics must be considered in the development of a treatment plan^66^.

## Conclusion

In regard to the differences in pediatric cases of COVID-19, children with and without CKD generally develop mild disease and do not require hospitalization in an intensive care unit. The papers reviewed in this study did not show significant differences in the severity of COVID-19 in these groups of patients. Therefore, current evidence does not allow the establishment of a relationship between CKD and severity of COVID-19 in children. Additional studies enrolling larger populations are needed.

Albeit rare, some children developed MIS-C as a possible complication of infection by SARS-CoV-2 and had more severe disease. This complication is apparently tied to decreased membrane-bound ACE2 expression. Therefore, studies looking into the polymorphisms of the ACE2 gene and expression levels may be usefu^23^, in defining disease severity and providing input to patient management.

Finally, adults with CKD had more severe COVID-19 symptoms compared to children with CKD. The underlying mechanisms that explain these differences are still the fruit of speculation, but they are possibly related to negative regulation of the ACE2/Ang-(1-7) axis in the elderly, greater expression of ACE2 in children, and differences in the immune response of pediatric, adult, and elderly patients. However, additional studies are required to confirm the ideas mentioned above.
